# Molecular Epidemiology of a *Pseudomonas aeruginosa* Hospital Outbreak Driven by a Contaminated Disinfectant-Soap Dispenser

**DOI:** 10.1371/journal.pone.0017064

**Published:** 2011-02-16

**Authors:** Simone Lanini, Silvia D'Arezzo, Vincenzo Puro, Lorena Martini, Francesco Imperi, Pierluca Piselli, Marco Montanaro, Simonetta Paoletti, Paolo Visca, Giuseppe Ippolito

**Affiliations:** 1 National Institute for Infectious Diseases “Lazzaro Spallanzani”, I.R.C.C.S., Rome, Italy; 2 Department of Biology, University “Roma Tre”, Rome, Italy; 3 UOC Ematologia Azienda Unità Sanitaria Locale di Viterbo, Viterbo, Italy; National Institute for Infectious Diseases (L. Spallanzani), Italy

## Abstract

**Background and Objective:**

*Pseudomonas aeruginosa* infection represents a main cause of morbidity and mortality among immunocompromised patients. This study describes a fatal epidemic of *P. aeruginosa* that occurred in a hematology unit in Italy.

**Methods:**

Retrospective cohort study, prospective surveillance, auditing, extensive testing on healthcare workers and environmental investigation were performed to define the dynamics and potential causes of transmission. RAPD, macrorestriction analyses and sequence typing were used to define relationships between *P. aeruginosa* isolates.

**Results:**

Eighteen cases of infection were identified in the different phases of the investigation. Of these, five constitute a significant molecular cluster of infection. A *P. aeruginosa* strain with the same genetic fingerprint and sequence type (ST175) as clinical isolates strain was also isolated from a heavily contaminated triclosan soap dispenser.

**Discussion and Conclusions:**

Our results are consistent with the hypothesis that patients became indirectly infected, e.g., during central venous catheter handling through contaminated items, and that the triclosan soap dispenser acted as a common continuous source of *P. aeruginosa* infection. Since *P. aeruginosa* is intrinsically unsusceptible to triclosan, the use of triclosan-based disinfectant formulations should be avoided in those healthcare settings hosting patients at high risk of *P. aeruginosa* infection.

## Introduction

The Gram-negative bacterium *Pseudomonas aeruginosa* (*P. aeruginosa*) is frequently associated with hospital acquired infections. While the nutritional versatility enables *P. aeruginosa* to occupy a variety of ecological niches in healthcare settings (HCS), its powerful armamentarium of virulence factors makes it highly pathogenic in susceptible patients [1]. *P. aeruginosa* is capable of causing high morbidity and mortality among immunocompromised patients [2], and it is a well-known cause of outbreaks in oncohematology units [3]–[4].

Patient-to-patient transmission through contaminated medical devices [5]–[7] and multi-vials drugs [8] is a well-established mechanism of *P. aeruginosa* spreading in HCS. Some studies have also emphasized the importance of the moist environment as a reservoir of nosocomial *P. aeruginosa* strains [1], [9]. Moreover, the resistance of *P. aeruginosa* to a variety of chemical compounds, including antibiotics, detergents, and hospital disinfectants facilitates its long-term persistence in the HCS and the spreading among patients [1], [6], [9]–[12].

During the first week of June 2007, two patients died in a oncohematology unit which had been opened since March 2006. The patients were severely immunocompromised as the consequence of chemotherapy for hematologic neoplasms. Clinical and laboratory findings were consistent with fatal systemic infections caused by *P. aeruginosa* strains sharing the same pattern of resistance to cephalosporins, fluoroquinolones and aminoglycosides. To prevent further spreading of infections, on 6 June 2007 the local health authorities decided to close the unit and to transfer patients and clinical activities to another hospital building.

The present paper reports the epidemiological investigation to confirm the presence of the outbreak, to identify the source(s) of infection, and to elucidate the transmission pathway(s). The report has been written according to “The ORION statement: guidelines for transparent reporting of outbreak reports and intervention studies of nosocomial infection” [13].

## Methods

### Ethical statement

All data contained in the manuscript are obtained during the epidemiological investigation commended by the Latium Regional Heath Authority (RHA), in order to identify/contain an ongoing epidemic cluster, to provide recommendations, to prevent new outbreaks and to avert complications in infected subjects. For the purpose of the current publication there was no information that could identify the patient personally.

The approval of INMI Spallanzani's IRB was not required since we operated under emergency circumstance, in fact, the investigation was commended by the RHA to contain an ongoing outbreak among frail subjects. Individual written consents were not obtained because of emergency circumstance (i.e.: potential risk of death of already infected subjects due to complications and the risk of further spreading of the infection) and because patients never underwent individual intervention for the purposes of this study but only according to their needs and clinical judgment.

### Study design

Table 1 summarizes the timeline of the individual tasks of the investigation, this includes: retrospective study, prospective surveillance, look-back investigation, test on healthcare workers (HCW), audit on infection control measures and environmental investigation.

**Table 1:** Time-line of the tasks.

Task	Date	Type of activity
**Retrospective study**	Jun 7 to 21	*Clinical chart review and internal data base analysis. All patients who had been admitted between 15/03/2006 and 06/06/2007.*
**Look-back investigation**	Jun 7 to 21	*Contacting and testing for P. aeruginosa.* *All patients who had been admitted between 14/02/2007 and 06/06/2007.*
**Prospective surveillance**	Jun 7 to Oct 4	*Microbiologic and clinical surveillance.* *All patients admitted between 07/06/2007 and 27/09/2007.*
**Healthcare Worker Testing**	Jun 13 to Jul 19	*Testing for P. aeruginosa.* *Healthcare workers employed in the unit.*
**Environmental investigation**	Jun 27	*Visual inspection of the unit and collection of environmental samples.*
**Auditing**	Jul 2 to 31	*Review of internal protocols on infection control. Interview of personnel.*

For each of the task the time and type of activity have been specified.

#### Retrospective study

Between 7 and 21 June 2007, a retrospective study was carried out to confirm the presence of the outbreak and to assess potential risk factors. For this task we designed a retrospective dynamic cohort for multiple failure events including all patients admitted between 15 March 2006 and 6 June 2007. Patients were enrolled the day of the first admission and censored on 6 June 2007 or if they died. Enrolled patients were considered at risk only while in hospital, and up to the first evidence of *P. aeruginosa* infection which occurred in each one of the admission(s) they had. Patients' data about clinical presentation, age, gender, time and place of hospital admission, timing of central venous catheterization (CVC), timing of urinary catheterization, timing of parenteral feeding and hematopoietic stem cell transplantation (HSCT) were acquired from clinical charts.

Data about *P. aeruginosa* infections were obtained from the database which was part of the infection control system implemented in the unit. According to this system, all patients underwent routine microbiological sampling on admission (i.e.: swabs from nasal, pharyngeal and rectal mucosa, swab from central venous catheter insertion site, when present, and urine cultures); additional microbiological tests were also performed throughout the hospital stay, when clinically appropriate. All positive tests, including antibiotic resistance phenotypes, were systematically recorded.

We considered a failure event the microbiological evidence of *P. aeruginosa* in a subject who had resulted negative at the routine tests at the admission.

#### Prospective surveillance

The prospective surveillance was performed to confirm the end of the transmission chain. For this task we designed a prospective dynamic cohort for multiple failure events including all patients admitted to the new hematology unit between 7 June and 27 September 2007. All these patients were screened both at time of admission and discharge according to the same sampling scheme as the internal surveillance system (see above).

#### Look-back investigation

In order to identify asymptomatic cases who might have been missed in the retrospective study, between 7 and 21 June 2007 all patients who had been admitted between 14 February and 6 June 2007 (i.e. in the 16 weeks preceding the closure of the unit) were contacted to be tested with the same sampling scheme as the internal surveillance.

#### Tests on HCW

To explore the potential role of HCW in the transmission of the infection, the personnel of the unit was asked to undergo tests for *P. aeruginosa* colonization. Tests were performed between 13 June and 19 July 2007 and included nasal, ear, rectal and urethral swab (if male) or vaginal swab (if female).

#### Auditing

The audit on infection control measures was carried out on July 2007 to assess potential breaks in infection control measures which could have been occurred. In this occasion all internal protocols were assessed and nurses were interviewed on hand hygiene practices according to Centers for Disease Control and Prevention (CDC) guidelines [14]. In addition the medical head of the unit and the nurse coordinator were asked about the general running of the unit.

#### Environmental investigation

The environmental investigation was conducted on 27 June 2007 to evaluate role of the environment in the spreading of the infection. In this day the unit was inspected and potential *P. aeruginosa* reservoirs were sampled. Specimens were taken aseptically, using sterile gloves and vials or swabs, depending on the sample. All the samples collected were immediately shipped to INMI Spallanzani to be processed.

### Clinical setting

**Figure 1:**
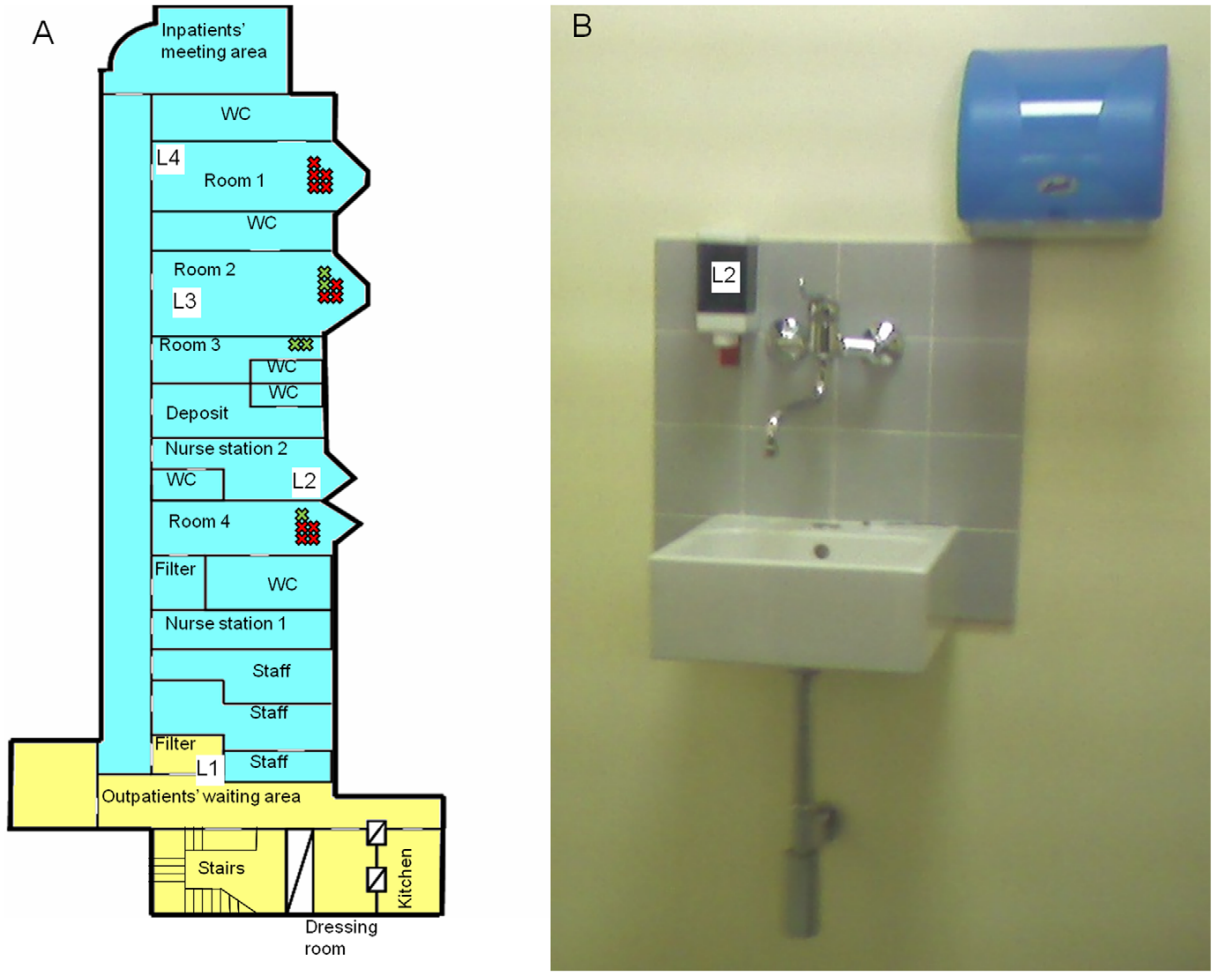
Hematology unit. (A) Map of the unit. Areas for outpatients and inpatients are in yellow and blue, respectively. Crosses indicate the location of patients with at least one *P. aeruginosa* isolate according to the case definitions (red, incident case; green, prevalent case; see text for details). L1 to L4 indicate the sites from which *Pseudomonas* spp. were isolated: L1, soap dispenser; L2 soap dispenser; L3 and L4 water outlets. (B) Drugs deposit and preparation room where the contaminated soap dispenser (L2) was placed.

The hematology unit comprised two different areas dedicated to outpatients and inpatients, separated by a filter room (figure 1A). The inpatients area consisted of 4 double rooms with in-side bathroom, 2 nurse stations where drugs (apart from antiblastics) and other medical devices were stored and prepared for use, a deposit for dirty materials, 3 rooms dedicated to doctors and nurses (staff area), and a meeting area for patients and visitors. The unit was staffed with 9 doctors, 15 nurses, and 3 auxiliary workers. Twelve nurses were dedicated to inpatients care, and 3 to outpatients.

### Case definitions

The following case definitions were used: (*i*) index case: the subject who introduced the epidemic strain in the unit; (*ii*) susceptible subject: a patient who was admitted without evidence of *P. aeruginosa* at admission tests; (*iii*) prevalent case: a patient who was admitted with evidence of *P. aeruginosa*; (*iv*) incident case: a susceptible subject who became positive for *P. aeruginosa* while in hospital; (*v*) probable case: an incident case infected with *P. aeruginosa* with an antibiotic resistance phenotypes identical to other confirmed cases and temporally related with other incident cases; (*vi*) confirmed case: an incident case infected with a molecular type of *P. aeruginosa* identical to another strain retrieved from the environment or from another incident or prevalent case; (*vi*) excluded case: an incident case which does not meet with any of the above definitions.

### Outcomes

For the purpose of this investigation we do not make distinction between colonized and infected symptomatic patients. Infection outcomes were: (*i*) survived: a cases who was still alive at the end of follow-up or had died without evidence of *P. aeruginosa* infection; (*ii*) died: a cases who died while infected and whose death was attributable to *P. aeruginosa* infection.

### Bacterial cultures

Microbiological culture tests on human samples were performed at local level, and the results were communicated as soon as available to the epidemiological team by the head of the unit. Identification of isolates and antibiotic susceptibility tests were performed with autoSCAN-W/A (W/A; Dade Behring Microscan Inc., West Sacramento, Calif.) [15].

Positive cultures were sent to the INMI Spallanzani for further analysis by means of the VITEK 2 system equipped with GN and AST-N022 cards (Bio-Mérieux). Blood cultures were analyzed with BacT/ALERT FN (Biomerieux). Search for *P. aeruginosa* in environmental samples was performed at INMI Spallanzani only by plating onto selective cetrimide (Pseudosel, Becton Dickinson) agar plates. Swabs of environmental surfaces were directly plated; water samples were concentrated on a 0.22 µm pore size filter (Millipore), suspended in saline and plated for *P. aeruginosa* counting; other liquid samples were appropriately diluted prior to plating. Bacterial growth was assessed after 24 and 48 h incubation at 37°C on Pseudosel agar, and expressed as colony forming units (CFU).

VITEK 2 numerical identification codes, hemolysis on Columbia-5% sheep blood agar plates, production of the fluorescent pyoverdine pigment on Pseudosel agar, and antibiotic-resistance (R-) profiles, were used for preliminary phenotypic analysis of *P. aeruginosa* isolates. Resistance to cefepime, ciprofloxacin, and gentamicin was used as marker of resistance to cephalosporins, fluoroquinolones and aminoglycosides.

### Epidemiological typing

In order to identify a confirmed case, the clonal relationship between *P. aeruginosa* isolates were assessed by means of Random Amplified Polymorphic DNA (RAPD) analysis, macrorestriction analysis by PFGE, and Multilocus Sequence Typing (MLST).

The RADP analysis was performed as a rapid screening test in order to provide the epidemiological team with information in the early steps of the investigation. The macrorestriction analysis, which is more reliable but also much more time-consuming, was eventually used to confirm RAPD data. The MLST analysis was performed to assign a genotype (ST) to the *P. aeruginosa* strains and correlate it to the population structure of the species.

RAPD reactions were performed in a volume of 25 µl with 40 pmol each oligonucleotide and 40 ng genomic DNA, using the Ready-To-Go PCR beads (Amersham). Oligonucleotide designations and their 5′-3′sequences are: 208, ACGGCCGACC; 228, GCTGGGCCGA; 241, GCCCGAGCGG; 272, AGCGGGCCAA; 275, CCGGGCAAGC; 277, AGGAAGGTGC; 287, CGAACGGCGG ERIC 1, ATGTAAGCTCCTGGGGATTCAC [16]–[18]. All PCR amplifications were carried out in a 9600 DNA Thermal Cycler (Perkin Elmer) under standard conditions [16]–[18]. Amplicons were separated by 1.5% agarose gel (Fisher) electrophoresis, stained with ethidium bromide, and photographed under UV transillumination. The 100-bp ladder (Biorad) was used as a molecular size standard.

Pulsed-field gel electrophoresis (PFGE) was carried out following the Health Protection Agency protocol (http://www.hpa.org.uk/), using a CHEF mapper (Bio-Rad, Segrate, Milan, Italy). PFGE profiles were interpreted according to published criteria [19], with a difference of four bands or less used to define epidemiological relatedness.

Electrophoretic profiles were analysed with Bionumerics software (Applied Maths, Sint-Martems-Latem, Belgium). The BioNumerics analysis was performed using the Dice coefficient and the unweighted pair group method of averages (UPGMA) with a 1% tolerance limit and 1% optimisation.

MLST was performed according to the reference protocol with sequencing primers for the housekeeping genes *acsA*, *aroE*, *guaA*, *mutL*, *nuoD*, *ppsA*, and *trpE*, under previously described PCR conditions [20]. Amplicons were purified by the QIAquick PCR kit (Qiagen). Sequencing reactions were performed using the BigDye Terminator kit, and products were run in an ABI PRISM 3100 Genetic Analyzer (Applied Biosystem). Nucleotide sequences were assembled for both strands and compared with existing entries in the MLST database (http://pubmlst.org/) for generation of allelic numbers and assignment sequence types (STs).

### Statistical analysis

The time of retrospective cohort study (15 March 2006 – 6 June 2007) was divided into sixteen 28-day fractions (T1–T16) to define the epidemic curve and calculate incidence rates with 95% confidence intervals (95% CI).

Extended Poisson model [21] for recurrent events with gamma distribution for inter-individual heterogeneity (gamma-frailty model) was used to assess the association of potential risk factors and the infection both in univariate and multivariate models. In univariate analysis we estimated incidence rate ratio (IRR) with 95% CI and Wald's test p-value for the association of infection with all potential risk factors. Multivariate model was set up including variables with IRR change ≥15% either as overall for binary variable (i.e.: HSCT and gender) or after 10 exposure units for continuous variables (i.e.: age, CVC, timing of urinary catheterization and timing of parenteral feeding). Analysis was performed using Stata Statistical software, version 11.1 (StataCorp, Texas, USA).

## Results

### Retrospective Cohort study

During the time of retrospective cohort study (15 March 2006 – 6 June 2007) 78 patients were admitted for a total of 4592 person-day at risk, and 10 incident cases (table 2).

**Table 2:** Characteristics of patients included in the study.

	Retrospective cohort	Prospective surveillance
Time period	15 March 2006 to 6 June 2007	7 June 2007 to 27 Sept. 2007
Time at risk (day/person)	4592	403
No. of patients	78	29
Median age (IQR a)	65 (50–73)	66 (58–73)
Male/female ratio	1.47	1.98
Main pathology (%)	Cancer (86.0); others (14.0)	Cancer (87.9); others (12.1)
Median day of stay (range)	22 (1–128)	13 (1–97)
Admitted as prevalent cases	5	1

a IQR, interquartile range.

**Figure 2:**
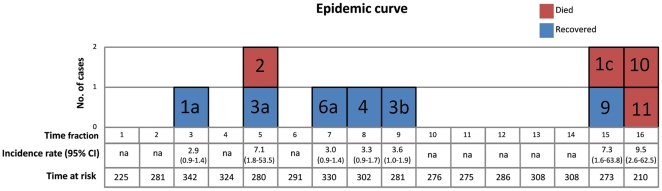
Epidemic curve. The diagram shows the 10 incident cases (with respective codes) identified throughout the 16 time fractions (T1–T16) of the retrospective cohort study. Red and blue squares denote died and survived patients, respectively. The incidence rate per 1000 person-days with 95% CI and the total time at risk is reported for each time fraction.

The epidemic curve and the incidence rates according to the 16 time fractions (figure 2) shows that *P. aeruginosa* infections clustered in two different time periods (T3–T9 and T15–T16), separated by a time period (T10–T14) with no cases.

The first cluster consisted of 6 incident cases (codes 1a, 2, 3a, 6a, 4, and 3b; figure 2 and table 3) one of whom died (code 2); *P. aeruginosa* strains isolated during this period showed heterogeneous antibiotic resistance phenotypes (table 3).

**Table 3:** Clinical and epidemiological features of 18 cases of *P. aeruinosa* infections identified throughout the investigation.

Summary of cases' clinical and epidemiological features											
Case code a	Sex/age	Diagnosis or symptom(s)	Outcome	Case definition	Positive specimens	Time fraction (room)	Phenotype				
							Cefepime	Ciprofloxacin	Gentamicin	Imipenem	Piperacillin
1a	Female/69	UTI	Survived	Excluded	Urine	T3 (Room 1)	R	R	R	S	S
					Blood		R	R	R	S	S
1b	Female/69	UTI	Survived	Excluded b	Urine	T4 (Room 3)	I	R	R	I	S
					Blood		I	R	R	S	S
2	Male/59	Septicaemia	Died	Excluded	Blood	T5 (Room 1)	R	R	R	S	R
3a	Male/66	Pneumonia	Survived	Excluded	Blood	T5 (Room1)	R	R	R	S	R
6a	Female/45	UTI	Survived	Excluded	Urine	T7 (Room 2)	I	R	R	S	S
4	Male/67	Vomit/diarrhoea	Survived	Excluded	Stool	T8 (Room 4)	I	R	R	S	S
5	Male/65	Fever	Survived	Excluded b	URT swab	T8 (Room 2)	S	I	S	R	S
6b	Female/45	Asymptomatic	Survived	Excluded b	Urine	T9 (Room 3)	I	R	R	S	S
3b	Male/66	Fever	Survived	Excluded	CVC swab	T9 (Room 4)	I	R	R	R	S
7	Male/32	Pneumonia	Survived	Excluded b	URT swab	T12 (Room 2)	S	I	S	R	S
8	Male/25	Asymptomatic	Survived	Index case b	Urine	T13 (Room 4)	I	R	R	S	S
9	Male/69	Pneumonia	Survived	Excluded	Sputum	T15 (Room 4)	S	R	R	S	S
1c	Female/70	Septicaemia	Died	Probable	Blood	T15 (Room 1)	I	R	R	S	S
10	Male/60	Pneumonia	Died	Confirmed	Sputum e	T16 (Room 1)	I	R	R	S	S
11	Female/66	Pneumonia	Died	Confirmed	Blood e	T16 (Room 2)	I	R	R	S	S
					Sputum f		I	R	R	S	S
12	Female/73	Asymptomatic	Survived	Probable	Rectal swab	T16 (Room 2)^d^	I	R	R	S	S
					URT swab		I	R	R	S	S
13 c	Male/52	Asymptomatic	Survived	Confirmed	Urethral swab	T16 (Room 4) d	I	R	R	S	S
					CVC swab e		I	R	R	S	S
14	Female/52	Asymptomatic	Survived	Excluded	URT swab	NA	S	S	S	S	S

UTI =  urinary tract infection; CVC =  central venous catheter; URT =  upper respiratory tract; S =  sensitive; I =  intermediate; R =  resistant; NA not applicable because the activity of the unit was moved to another hospital building.

^**a**^ Cases were coded by assigning to each patient a progressive number (i.e.: 1–14); subsequent cases in the same patient were identified by adding a letter (i.e.: a, b and c) to the patient's number.

^**b**^Prevalent cases.

^**c**^This patient was admitted to the new located unit at the time of the look-back therefore he is previously identified as an asymptomatic case in the look-back and eventually, as a prevalent case, in the prospective surveillance.

^**d**^Case identified during look back; for them time-fraction and room number is referred to their last admission.

**Figure 3:**
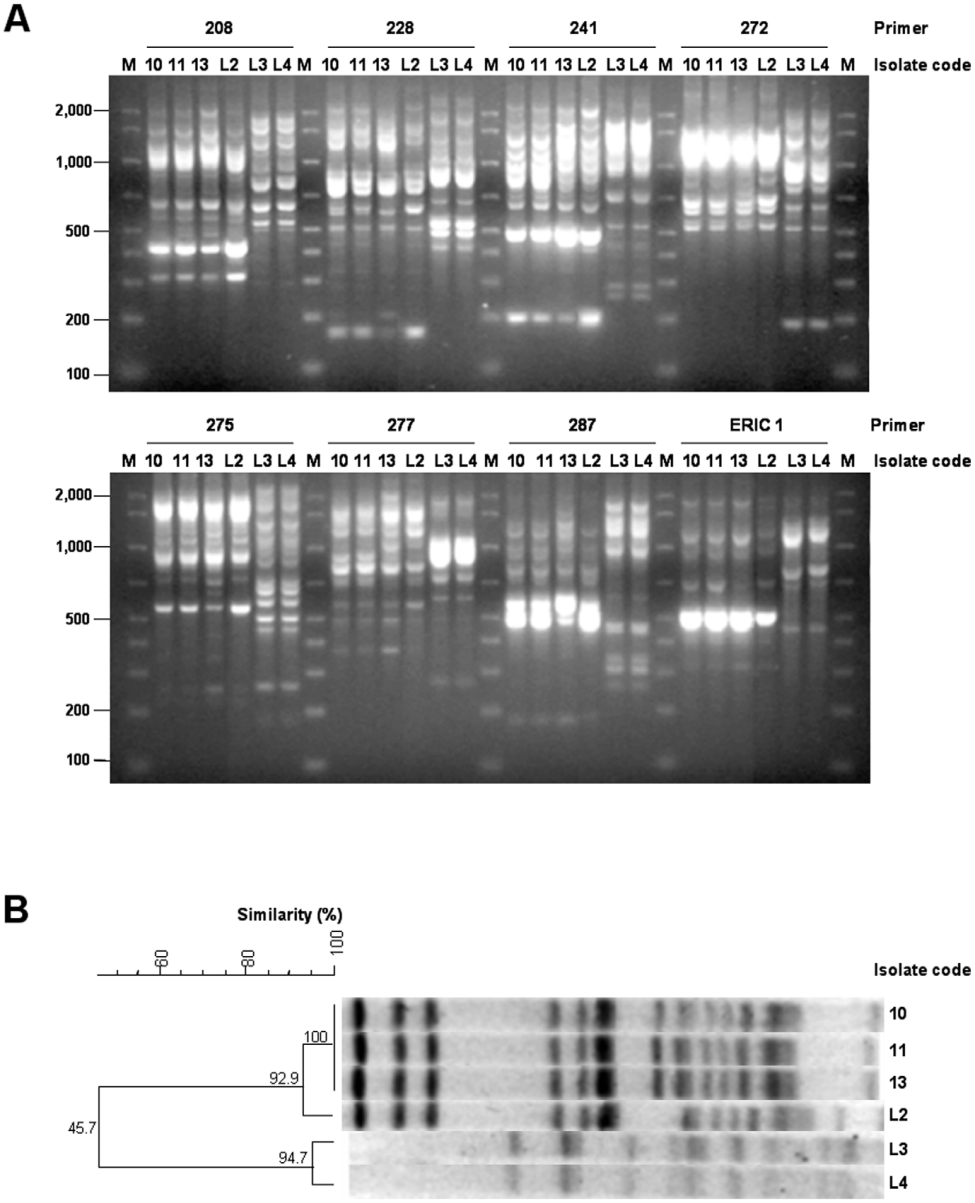
Epidemiological typing of clinical and environmental *P. aeruginosa* isolates. (A) RAPD analysis with primers 208, 228, 241, 272, 275, 277, 287 and ERIC 1, as indicated on top of electropherograms. (B) PFGE analysis of *P. aeruginosa* isolates. The dendrogram was generated with BioNumerics (Applied Maths) using the unweighted pair-group method with arithmetic averages (UPGMA) and the Dice coefficient. The similarity (%) between isolates is shown at each node of the dendrogram.M is the molecular weight marker (bp); 10 *P. aeruginosa* from sputum (patient 10); 11 *P. aeruginosa* from blood culture (patient 11); 13 *P. aeruginosa* from central venous catheter swab (patient 13); L2 *P. aeruginosa* from soap; L3 and L4 *P. aeruginosa* from water.

^**e**^These samples were used for bacterial typing as shown in fig. 3.

^**f**^This represent 2 specimens taken the same day and yielding an identical molecular type to that found in the blood culture (data not shown).

The second cluster (T15–T16) consisted of 4 incident cases (codes 9, 1c, 10, and 11; figure 2 and table 3). In contrast to the first cluster, *P. aeruginosa* infections which occurred in the second cluster were significantly more severe (3 patients died despite prompt empiric antibiotic therapy), and the 3 lethal isolates shared an identical antibiotics resistance phenotypes.

No spatial relations among cases were noticed in either period (figure 1A and table 3).

The univariate analysis of risk provided good evidence of association between infection and the duration of CVC (IRR after 10 days of catheterization  = 1.23; 95% CI 1.04–1.45; p = 0.014) and the duration of parenteral feeding (IRR after 10 days  = 1.36; 95% CI 1.04–1.79; p = 0.024). The multivariate model showed that only the duration of CVC was independently associated with the risk of infection (IRR after 10 days of catheterization  = 1.43; 95% CI 1.05–1.95; p = 0.022). Complete results of the risk analysis are reported in table 4.

**Table 4:** Association between *Pseudomonas aeruginosa* infection and selected characteristics.

Risk analysis				
	Univariate analysis		Multivariate analysis	
Risk factor	IRR (95% CI)	p-value	IRR (95% CI)	p-value
Female gender	0.87 (0.24–3.07)	0.826	-	-
Age in years at time of admission	1.35 (0.77–2.36)^a^	0.288	1.93 (0.86–4.32) a	0.109
Days of central venous catheterization	1.23 (1.04–1.45)^b^	0.014	1.43 (1.05–1.95) b	0.022
Days of parenteral feeding	1.36 (1.04–1.79) b	0.024	1.02 (0.69–1.52) b	0.904
HSCT in the previous years	2.33 (0.60–9.03)	0.219	3.91 (0.76–20.09)	0.102
Days of urinary catheterization	1.20 (0.78–1.84) b	0.412	0.82 (0.41–1.63) b	0.571

IRR =  incidence rate ratio; 95% CI = 95% confidence interval; HSCT =  hematopoietic stem cell transplant.

a IRR is reported for 10 years increment of age.

b IRR is reported for 10 days increment of exposure.

### Prospective surveillance

During the time of prospective surveillance (7 June – 27 September 2007) 29 patients were admitted for a total of 403 person-day at risk (table 2). Throughout this period 2 cases of infection were identified; one was a prevalent case (code 13 table 3) and the other was an incident case with a very different resistance phenotype from the cases previously occurred (excluded case; case 14 table 3).

### Look-back investigation

Sixteen out of 20 eligible subjects were tested for the look back analysis (4 patients were not available: 3 patients died and one did not accepted to be tested). Among these 16 we identified 2 patients who resulted positive to *P. aeruginosa* sharing identical resistance phenotype of the 3 lethal incident cases observed in the second infection cluster (table 3 cases 12 and 13); only one isolate from case 13 was available for molecular analysis.

### Tests on HCW

Tests for *P. aeruginosa* were done on 21 out of 24 HCWs, as 3 of them refused testing. None of the tested HCWs resulted positive.

### Audit on infection control measures

The audit of internal protocols showed good application of infection control measures. Ten of the 12 nurses who cared for in-patients were interviewed and reported good application of hand hygiene. In particular, a part from the absence of alcohol handrub, HCWs claimed to wash their hands with disinfectant soaps contained in common soap dispenser (either clorexidine digluconate 4% -*Neoxidina Mani®*- or triclosan 0.5% -*LH Crema Mani®*- used in rotation of 3 months, figure 1B), and wear a new pair of gloves each time they move to a new patient.

Environment cleaning protocols reported that all rooms were cleaned by an external team that was also responsible for refilling soap dispensers without direct supervision by HCWs.

When asked the nurse-coordinator and the head of the unit excluded that drugs contained in multi-dose vials could have ever been used.

### Environmental investigation

On 27 June 2007, the unit was inspected and potential environmental reservoirs were sampled. The inspection showed that the unit was provided with water outlets and with a common refillable soap dispensers (figure 1B). Environmental reservoir sampling consisted of 29 different specimens taken from opened disinfectant bottles, water outlets, sinks, soap dispensers, and showers.

### Microbiological investigation and *P. aeruginosa* typing

As shown in figure 1A (codes L1 to L4), of the 29 environmental specimens that were investigated for *P. aeruginosa* contamination, only 4 yielded visible growth on selective Pseudosel agar plates. Bacterial identification of representative colony morphotypes from each plate confirmed the presence of 5 different strains, i.e.: one *Pseudomonas stutzeri* from the soap dispenser in the filter room between the outpatient and inpatient area (code L1 in figure 1A); one *P. aeruginosa* from the soap dispenser in the nurse station where drugs and medical devices were also stored (code L2 in figure 1A and 1B); one *P. aeruginosa* from the water outlet in patients room 2 (code L3 in figure 1A); one *P. aeruginosa* and one *Pseudomonas mendocina* from the water outlet in patients room 1 (code L4 in figure 1A).

Bacterial counts revealed heavy contamination (ca. 5×10^4^ CFU/ml) of the soap sample (code L2 in figure 1A and 1B), and much lower levels (6-to-8 CFU/ml) in both water samples (codes L3 and L4 in figure 1A). The levels of *Pseudomonas stutzeri* in soap sample L1 (figure 1A) and *Pseudomonas mendocina* in water sample L4 (figure 1A) were ca. 10^3^ and 5×10^2^ CFU/ml, respectively.

The *P. aeruginosa* soap isolate (code L2 figure 1A and 1B) showed the same phenotypic traits as all clinical isolates (table 3 codes 10, 11 and 13); all of them were haemolytic, yellow-green fluorescent on Pseudosel agar, and showed similar multidrug-resistance pattern (resistant to gentamicin and ciprofloxacin, intermediate resistant to cefepime and susceptible to piperacillin and imipenem) and VITEK 2 identification codes. The two water isolates were also similar to each other, but differed from soap and clinical isolates for several phenotypic traits, namely, pigment production, antibiotic-resistance (sensible to gentamicin, ciprofloxacin, cefepime, piperacillin and imipenem) and VITEK 2 identification codes. *Pseudomonas* spp. other than *P. aeruginosa* were not further investigated.

The typing analysis was performed on *P. aeruginosa* isolates: *i.e. 5* clinical isolates from 3 patients (one blood culture and 2 sputum cultures from patient 11; one sputum culture and one culture from central venous catheter insertion site swab, from patient 10 and patient 13, respectively), and the 3 environmental *P. aeruginosa* strains.

Preliminary RAPD analysis with 8 different primers (figure 3A) showed genotypic identity between the soap isolate (code L2 figure in figure 3A) and available clinical strains (patient 10, 11 [blood culture; other not shown] and 13 in figure 3A), but remarkable differences between this group and the two water isolates (codes L3 and L4 in figure 3A). Likewise, the PFGE patterns of *Spe*I digested genomic DNA from clinical isolates 10, 11 and 13 (figure 3B) were identical to each other and closely related to the soap isolate L2 (2 bands difference; figure 3B). In contrast the isolates L3 and L4 from water (figure 3B) resulted unrelated to both clinical strains and soap isolate.

MLST was performed to assign them an univocal and shareable genotype (ST). Identical allelic numbers could be determined for individual gene sequences in all strains, which made it possible to assign the whole cluster to ST 175.

All the typing results concur to link the soap isolate L2 with clinical isolates 10, 11 and 13 (confirmed cases).

## Discussion

Based on the evidence collected along the investigation, it is likely that 3 out of 4 incident cases that occurred between T15 and T16 and the 2 cases identified during the look-back represent a sole epidemic cluster (i.e.: 3 confirmed and 2 probable cases) compatible with an environmental continuous common source of infection within the unit.

Several evidence support this hypothesis. Firstly, T15 and T16 showed the highest incidence ever in the unit, infections during this period were more severe than the previous ones and all the 5 cases were admitted in this period. Secondly, the retrospective analysis of microbiological data showed that these 5 cases were infected with bacterial strains sharing the same antibiotic resistance phenotype. Thirdly, the spatio-temporal distribution of cases was consistent with a common continuous source of infection which all patients were exposed to regardless their location in the unit and, in fact, no new case of infection due to the epidemic strain occurred when the patients were moved to a different hospital building.

The identification of the index case remains so far tentative. We believe that the most probable index case might have been a 25-year old man with acute myeloid leukemia and neurogenic bladder needing of intermittent catheterization who had been admitted as prevalent case (code 8 in table 3) one month before the onset of the first incident case; he might have contracted *P. aeruginosa*, in the form of asymptomatic bacteriuria, at home during the urinary catheterization, and eventually contaminated HCWs' hands or the environment while admitted.

Also the source of transmission was not definitively ascertained. The use of multi-vials drugs [8] was unlikely since both the nurse-coordinator and the head of the unit claimed that drugs in multi-dose vials were never in use and no hint of this practice was found through unit inspection and internal protocol review. Due to legal issues, the investigation team could enter the unit only 3 weeks after the closure. As *P. aeruginosa* can survive only in moist conditions, no efforts were spent for sampling desiccated surfaces such as doors, walls nearby the beds or trolleys. Therefore, it could not be determined whether or not those surfaces were contaminated during the outbreak and if they might have played a role in the transmission of the infection. Other potential environmental reservoirs such as water outlets [22] and plumbing fixtures (e.g. sinks and showers) [23] were excluded by the environmental investigation.

The environmental investigation found that one of the soap dispenser, where HCWs used to wash their hands, was heavily contaminated with the same molecular type of *P. aeruginosa* as confirmed cases which is compatible with an environmental common continuous source of infection as above hypothesized.

With regard to the pathway of transmission, considering all incident cases since the opening of the unit, a good evidence of association between infections and duration of CVC was found. This, along with previous findings, suggests that central venous catheter handling by transiently contaminated HCWs might have played a role in the spreading of *P. aeruginosa* during the epidemic period.

A similar pathway of transmission has been also reported in other published outbreaks related to soap dispenser contamination [3], [24], [25]. Thus it may be hypothesized that, also in this circumstance, the 5 clustered cases became infected through HCWs' hands contaminated during hand washing using the soiled soap dispenser which acted as a continuous source of *P. aeruginosa*. Indeed, the reported optimal adherence to hand-washing practice by HCWs may have even facilitated the spreading of *P. aeruginosa*. Table 5 repots overall epidemiological outcomes at the end of the outbreak according to the above hypotheses.

**Table 5:** Overall epidemiological outcomes at the end of the outbreak, according to the assumption that case 8 was the index case and the soap dispenser the actual environmental reservoir.

Summary of the epidemiologic parameters	
Epidemic period	08/03/2007–06/06/2007
No. of exposed susceptible	19
No. of cases	5 (3 confirmed, 2 probable)
No. of fatalities	3
Attack rate (%)	26.3
Fatality rate (%)	60
Source	Soap dispenser
Associated factors	CVC

The specific way by which the soap dispenser became contaminated is uncertain. We hypothesize that some breaks in cleaning the soap dispenser before refilling it by the external team may have been an important factor in determining contamination and pathogen growth, possibly facilitated by the suboptimal bactericidal activities of triclosan against *P. aeruginosa* [26]. Therefore we recommended to use safer soap-dispensers, such as those provided with disposable not refillable cartridges with anti-reflux valve, and to consider disinfectant soaps with highest bactericidal activity such as clorexidine-based soap. Moreover, we advised to implement the use of alcohol-based handrub, when appropriate, as recommended by current international guidelines [27]. The new located unit complied with all these recommendations.

In this study, molecular typing was essential to link clinical *P. aeruginosa* isolates (codes 10, 11, and 13) with each other and with the isolate from the soap dispenser (L2) and ruling out any correlation with the other environmental isolates (L3 and L4). Notably, such a link was preliminarily inferred by the results of simple phenotypic tests, reviving the old notion that the examination of phenotypic characters, such as antibiograms, may help in the preliminary identification of an outbreak strain at the local clinical laboratory level.

The association of *P. aeruginosa* ST 175 with an outbreak of severe infections resulting from contamination of the hospital environment highlights the importance of this ST as a nosocomial pathogen. ST 175 does not belong to any of the main *P. aeruginosa* clonal complexes, although pathogenic strains with this type have previously been isolated from the sputum of cystic fibrosis patients in Canada and UK, and from Intensive Care Units in Poland (http://pubmlst.org/perl/mlstdbnet/mlstdbnet.pl?file=pa_profiles.xml) [28]. The high virulence potential of our ST 175 isolate is inferred by the rapid fatal outcome of infection observed for two of three confirmed cases, despite the prompt initiation of empirical antibiotic therapy.

Potential limitations of this study include: (*i*) only a limited number of cases was investigated by molecular typing due to the lack of preserved samples, which prevent us to clearly define the index case; (*ii*) it is possible that the number of incident asymptomatic cases of infections were underestimated, given the design of the internal surveillance protocol; (*iii*) since the unit was closed to prevent further spreading of infection, an observation-based assessment of working procedures could not be performed, therefore we cannot provide a more comprehensive description of critical aspects of assistance; (*iv*) it could not be established the potential role desiccated surfaces in the spreading of the infection.

Despite these limitations, this investigation highlights some topical issues. First, prospective surveillance of emerging hospital pathogens, such as *P. aeruginosa*, proved to be an effective measure for prompt identification and containment of ongoing epidemic clusters, as demonstrate by the small number of cases and short duration of this outbreak. Second, this study highlights the synergistic power of combining classical epidemiology with molecular typing in tracing back the source of *P. aruginosa* infection and defining its transmission pathways, also in cases of small outbreaks and unprecedented environmental source. Third, the outbreak strain was assigned to an uncommon sequence type (ST175), which we showed to be highly pathogenic even among non-cystic fibrosis patients.

We believe that researchers should be encouraged to publish outbreak reports in HCS. In fact, as other observational studies, outbreak reports may contribute to a better understating of infection spreading and dynamics in HCS, and provide essential information to compare different approaches for preventing new events and managing ongoing ones. The increasing interest for outbreak investigations in HCS is confirmed by: (*i*) the implementation of open-source databases dedicated to HCS outbreaks only [29]; (*ii*) the implementation of specific guidelines for reporting HCS outbreaks [13]; (*iii*) the increasing number of systematic reviews and/or meta-analyses which use outbreak investigations as source of data.

*P. aeruginosa* outbreaks among immunocompromised patients represent dangerous events and they may recognize unusual pathways of transmission, such as contamination of disinfectants [3], [30]–[31]. In order to avoid the occurrence of new event additional studies are needed to define the susceptibility of *P. aeruginosa* strains to commonly used disinfectants, in particular triclosan, with special regard to those used in hospital units which admit frail patients, such as oncohematology units.
